# Supplementary material for: Stakeholder Perspectives on the Development and Implementation of a Polypharmacy Management Program in Germany: Results of a Qualitative Study
Source: J Pers Med. 2022 Jul 7;12(7):1115. doi: 10.3390/jpm12071115 (PMC9319191; doi:10.3390/jpm12071115)
Supplement: Supplementary file 1 [file jpm-12-01115-s001.zip › Supplementary Material/Supplementary Table S2_Interview Guide.pdf]

Subject	Questions	Follow-up questions	Positive reinforcement	Notes
Greeting and introduction	Hello. My name is … Thank you for agreeing to be interviewed. The interview is likely to take around 60 minutes.			
Description of the research project	As you will have seen from the E-Mail, I would like to ask you some questions as part of the EVITA study entitled “Evidence-based Polypharmacy Program with Implementation in Health Care”, which is being sponsored by the German Innovation Fund. Do you have any questions before we begin?			
Consent	We have already informed you that today’s interview will be recorded on an audio recording device. Is that OK for you? All content will of course be pseudonymized, and it will not be possible for third persons to identify you.		I would like to ask you to take the time to consider your answers. We have plenty of time for you to pause for thought.	
Return to what has already been discussed	I would like to talk to you about the health care of persons with multimorbidity and polypharmacy. When you think of persons with multimorbidity and polypharmacy, what groups of people come to mind?		Age groups, severity, functional limitations, not only the number of diseases/drugs, heterogeneous target group	
Introductory question	As an expert * and/or representative* of XXX, please tell me what you think about the current healthcare of persons with multimorbidity and polypharmacy in Germany.	* What special challenges do you see in providing care to persons with multimorbidity and polypharmacy?<br>*What do you view as particularly positive about the current healthcare of persons with MM/PP?<br>* What do you view as particularly negative about the healthcare of persons with MM/PP?<br>*In your view, what is it about the current situation that should change?<br>*Why do you think that?<br>*How could things change/what would it take to make the changes?	I can understand that. Would you like to go into more detail?	

Subject	Questions	Follow-up questions	Positive reinforcement	Notes
Transition	Structured care programs, so-called DMPs exist in Germany for chronic diseases like diabetes. What would you say to the development of such programs for persons with MM/PP?	*MM+PP affect persons with chronic diseases. Several DMPs might be applicable to one patient. New Multimorbidity Management Program that would not be disease-based.		
Specific questions	What groups of patients do you think should be offered the chance to participate in a structured management program for MM/PP?	If you were asked to name three criteria for inclusion in such a structured care program, what would immediately come to mind?<br>Who should not be considered for inclusion?		
Specific questions	What do you think should be the individual components of such a program?	*What interventions do you consider important/useful?<br>*Why did you mention those in particular?		
Specific questions	In view of the fact that various professional groups cooperate in providing healthcare, which groups do you think should be included in such a structured program?	*Why did you mention those in particular?<br>*Could you imagine that work could be divided up better by, for example, delegating certain tasks? If so, to whom?<br>*What should be taken into consideration when doing that?<br>*What tasks could be delegated?<br>*And who else should be involved?	Consider returning to the topic if certain professional groups are not mentioned (GP, Specialist, Pharmacist, medication coach (drug safety advice provided by health funds)	
If not yet mentioned<br><br>Specific questions (Implementation)	Up to now, we've been speaking about the content of such a program. How could such a structured treatment program be implemented in routine care?	*Do you think the regulatory framework, compensation and other such issues would have to change fundamentally, in order to provide improved healthcare to these patients?<br>*What changes do you think would be necessary?<br>*What existing elements could be built upon?<br>* What factors do you think would hinder the introduction of such a structured care program?<br>* What factors do you think would promote and support the introduction of such a structured care program?		

Subject	**Questions**	**Follow-up questions**	**Positive reinforcement**	**Notes**
Specific questions	What effects do you think would result from the introduction of such a care program?	*And for whom (patient? provider?)<br>* Assuming such a program existed, what do you think would be the advantages/disadvantages for you and your institution?<br>*(Could you imagine such a program would have any unintended consequences?)	*System level, Individual level, Practice level…<br>*What persons/participants/ structures could you imagine?	
Specific questions	What role could your own profession/institution play in the development of such a care program and its implementation in routine care?			
Additional questions	Who else do you think we should discuss this topic with?			
Additional questions	Is there anything else you would like to tell us?			
Leave-taking	Thank you very much for the discussion. Your answers will certainly help us. If you think of anything else that is important, please do not hesitate to send us an E-Mail during the next week.			
